# Goal Format in Small-Sided Soccer Games: Technical Actions and Offensive Scenarios of Prepubescent Players

**DOI:** 10.3390/sports4040053

**Published:** 2016-11-25

**Authors:** Craig Pulling, Alex Twitchen, Carl Pettefer

**Affiliations:** Chichester Institute of Sport, University of Chichester, Chichester PO19 6PE, UK; a.twitchen@chi.ac.uk (A.T.); CPETTEF1@chialumni.ac.uk (C.P.)

**Keywords:** technical component, player development, performance analysis, tactical component, prepubescent soccer

## Abstract

The aim of this study was to investigate the influence of the number of goal-posts and the positioning of goal-posts used within small-sided games on the frequency of technical actions and offensive scenarios performed by prepubescent players within soccer. The participants were eight male prepubescent soccer players (12.1 ± 0.5 years). The participants were video recorded for 20 min playing four different formats of 4v4 small-sided games: (1) standard two goal game; (2) four goal game, one goal in each corner; (3) two goal game with goal-posts positioned 9.14 m/10 yd infield, scoring only through the back of the goal; (4) four goal-game, one goal positioned 9.14 m/10 yd infield in each corner, scoring through either the front or back of each goal. Chi-squared tests of independence were utilized to statistically explore the impact of the different small-sided game formats. There were significant associations (*p* < 0.05) observed between the different small-sided game formats and the frequency of turns, dribbles, shots, goals and overlaps performed. For example, players performed more turns in small-sided game format two and more shots during small-sided game format four. It is suggested coaches should consider using a variation of the number and positioning of goal-posts in small-sided games as an effective training tool in the development of prepubescent soccer players. This will enable coaches to vary the focus of sessions, and develop specific technical and tactical actions within a situation similar to that of real match-play.

## 1. Introduction

As soccer is one of the most popular team sports in the world, there has been a considerable amount of research on soccer coaching methods that may facilitate the acquisition of skilled performance [1,2,3]. Successful performance within soccer is reliant upon psychological factors, technical and tactical skills, and the physiological capabilities of an individual [4]. Technical actions in soccer are the individual skills that are performed by the players such as passes and shots [5]. Whereas tactical actions involve the players applying offensive principles of play when their team is in possession of the ball (examples include width, depth and penetration) and defensive principles when their team is not in possession (examples include delay, balance and cover) [6].

Within soccer, a popular strategy that many coaches incorporate into training programmes is the use of small-sided games (SSG) [7]. SSG are increasingly being used as a specific training tool in the development of young soccer players [8]. They provide a practice environment where players will experience situations that closely resemble real match-play and, therefore the influence of SSG format on technical and tactical actions is particularly relevant when planning soccer training. Duarte et al. [9] concluded that the use of SSG within soccer training is an efficient strategy to increase a player’s specific practice time (deliberate practice), consequently improving technical skills within a tactical and decision-making environment. Possessing knowledge of a particular SSG format that naturally encourages players to use the technical skills more frequently would be useful information for a coach.

**Figure 1:**
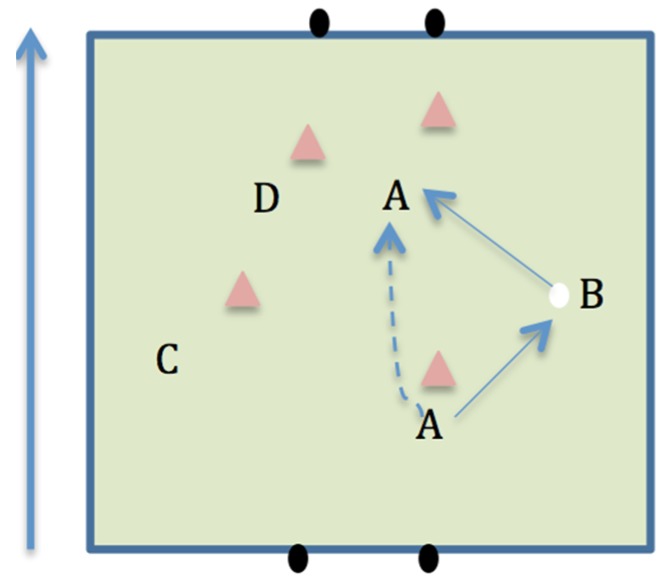
Player A performing a one-two.

**Figure 2:**
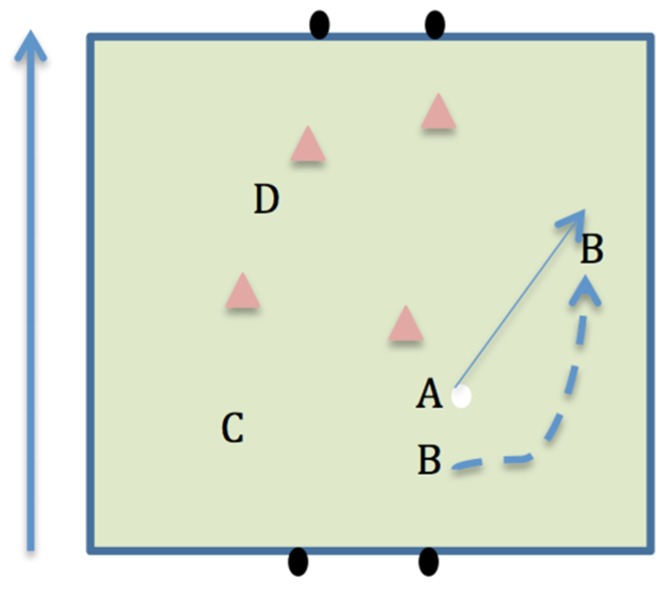
Player B performing an overlap.

Individual and collective competence when performing offensive scenarios can aid successful performance in soccer [10]. Offensive scenarios include: 1v1; one-two’s (around a defender, see Figure 1); and overlapping runs (see Figure 2). Many coaches would typically associate 1v1 scenarios in soccer with wingers attacking fullbacks. However, Hulln [11] argued that the game of soccer is a continual transition of 1v1 confrontations. For example, if a winger fails in an attempt to dribble the ball past the fullback, they may pass to a central midfielder, who is then in a 1v1 situation with the opposing midfielder. It could be argued that a SSG format that naturally produces more 1v1 scenarios would be beneficial for player development. The one-two in soccer is used to break down opposition defences and commit defenders, allowing attacking players to receive the ball in more advanced positions. Bauer [12] stated that the effective use of the one-two may result in the defensive players being more reluctant to rush in and tackle, subsequently allowing more space for the attacking team to play. Overlapping runs within soccer increase the number of players attacking the goal and can increase a team’s chance of scoring [13]. They also create a distraction for the defender, who is forced to make a decision of which player to mark (the player in possession of the ball or the player making the overlapping run). This defensive uncertainty can gain an advantage for the attacking team [14]. SSG provide a practice environment where players have the opportunities to experience defensive and offensive scenarios [6].

Previous research within SSG in soccer has focused on the effects of manipulating factors such as pitch dimensions [15,16,17] and the number of players [18,19,20,21], as well as exploring physiological responses [22,23] and conducting time motion analyses [24,25]. Previous research that has investigated the scoring mode in SSG has investigated fluctuations in player heart rate [26]; physiological, physical and technical performance [27]; tactical behaviour [28]; and defensive performance [7]. Travassos et al. [28] concluded that future research should investigate the manipulation of the number of goals used in SSG. The aim of this study was to investigate the influence of the number of goal-posts and the positioning of goal-posts used within small-sided games on the frequency of technical actions and offensive scenarios performed by prepubescent players within soccer. It is hypothesized that different formats of SSG will lead to different frequencies of technical actions and offensive scenarios being performed by prepubescent soccer players.

## 2. Methods

### 2.1. Participants

The participants were eight male prepubescent soccer players. The mean (±SD) age of the participants was 12.1 (±0.5) years. The participants were recruited from a local secondary school and they were all members of the school soccer team. At the time of testing, all participants played soccer for local community soccer clubs, with two of the participants also attending weekly training sessions with a professional soccer club academy. The mean (±SD) amount of deliberate practice experience of the participants in soccer was 5.4 (±1.0) years. All participants were free from injury at the time of testing. The parents of the participants were provided with an information sheet that clearly detailed the procedures involved in the study and the perceived benefits and risks to the participants. Informed consent was obtained from parents of the participants prior to any data collection. Ethical approval was granted from the university’s research ethics committee prior to data collection.

### 2.2. Procedure

**Figure 3:**
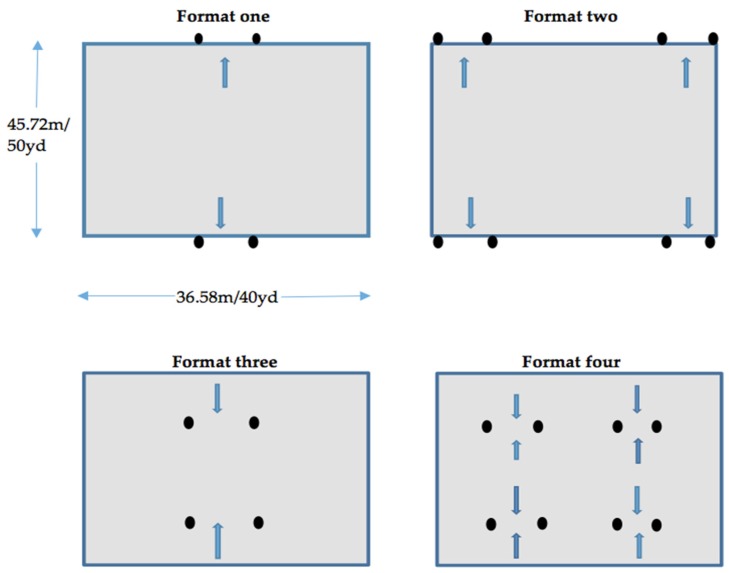
Small-sided games (SSG) formats.

All data was collected over a period of four weeks. Four formats of SSG were applied (see Figure 3). Format one was a standard 4v4 game with two goal-posts. A goal is scored by passing/shooting the ball through the goal. Format two was a 4v4 game with four goals. One team attacks two end goals, the other team attacks the other two end goals. A goal is scored by passing/shooting the ball through either goal. Format three was a 4v4 game with two goals. The goals were positioned 9.14 m/10 yd infield from the end line. A goal is scored by passing/shooting the ball through the back of the goal. Format four was a 4v4 game with four goals. The goals were positioned 9.14 m/10 yd infield from the end line. One team attacks two end goals, the other team attacks the other two end goals. A goal is scored by passing/shooting the ball through either the front or back of the two end goals. The same rules applied for all SSG formats other than how a goal is scored. After a goal was scored, the team who conceded the goal had a free pass from the end line. There were no throw-ins during the SSG; play was restarted from the side-line with a free pass from where the ball left the pitch.

For each SSG format, the same size pitch was used (45.72 m/50 yd × 36.58 m/40 yd), with all goals being 1.83 m/2 yd wide. It was decided this was an appropriate pitch size in line with the English Football Association [29] recommended pitch sizes for 9v9 U-12 soccer (73.15 m/80 yd × 45.72 m/50 yd). Due to the absence of goalkeepers in all four of the SSG formats, a 45.72 m/50 yd × 36.58 m/40 yd area created the same pitch area per outfield player ratio (209 m^2^/250 yd^2^) as 9v9 prepubescent matches. All the SSG were conducted on a third generation artificial turf surface.

For all SSG data collection, the same eight participants were used and they performed the same warm-up routine each week. The participants were informed of the game duration and rest periods prior to participating. Each week 2 × 10 min games were played for one of the four formats of the SSG. The order of the SSG formats was established randomly using a random number generator. Each 10 min game constituted 2 × 5 min halves with a three minute break between each half. Players were then given a further five minute rest period before engaging in a second 10 min game using the same structure as game one [7]. Other than the SSG format (amount and positioning of goal-posts), all other variables including pitch dimensions, number of players on each team, and recovery periods remained constant. This is supported by Aguiar et al. [30] who concluded that the use of standardized conditions in SSG studies would probably allow for a better understanding about the role of individual factors that may help researchers to find more reliable conclusions. For each SSG format the rules of the game were explained to the participants and then the participants played freely with no coaching input. The SSG were recorded using a video camcorder (Panasonic HDC-HS60 Hi-Def). The video camcorder was positioned 10m from the side-line and perpendicular to the field of play. This set up enabled the entire playing area to be captured, yet still allow for a precise analysis of the action variables.

With the support of prior studies [31,32], clear and concise operational definitions were devised (see Table 1) between the analyst (a former professional soccer player with over 10 years’ experience at professional level) and a university senior lecturer who has over 5 years’ experience of analyzing soccer. To check the practicality of the operational definitions, pilot footage [33] was then used to ensure the clarity and ease of recognition of the action variables fundamental to the current study [28].

### 2.3. Reliability

In the current study both inter-observer and intra-observer tests were conducted on 10 min of randomly selected SSG footage. This equated to 12.5% of the total video footage recorded. Reliability was calculated using the Kappa statistic. The inter-observer reliability test involved a physical education teacher with over 10 years’ experience in the profession. The physical education teacher was provided with the operational definitions and given a 15 min training session on how to analyze the footage. The intra-observer reliability test was conducted three weeks following the initial analysis to reduce the potential of learning effects. The inter-observer Kappa statistic was 0.83, whilst the intra-observer Kappa statistic was 0.87.

### 2.4. Data Analysis

All data are presented as absolute frequencies. Successful and unsuccessful passes forwards, sideways, and backwards and penetrating passes are also presented as a percentage (stated in parentheses) of the total number of passes within each SSG format. Passing success was explored statistically across the different SSG formats by applying a two-way chi-squared test of independence. For all other action variables (turning, dribbling, shot, goal, overlap, one-two, 1v1) a one sample chi-squared test of independence was used to statistically explore the impact of the different SSG formats. The chi-squared test of independence examines the association between two variables according to the distribution of the frequencies. The alpha level was set at 0.05. A limitation of the one sample chi-squared test is that it is not capable of measuring the size of the statistical difference between the different SSG formats.

## 3. Results

For all four SSG formats the players were more likely to pass the ball successfully and maintain possession of the ball than to unsuccessfully pass the ball. There were 803 passes attempted across the four SSG formats, with format one having the most passes attempted (232 passes) and format four having the least passes performed (168 passes). Format three had the highest percentage of successful passes (85.2%), whereas format four had the lowest percentage of successful passes (75.0%). However, there was no significant association between the SSG format and passing success (χ2(3) = 6.69, *p* > 0.05) (Table 2).

The players performed the greatest amount of turns when playing SSG format two (45), whilst turning was not frequently performed during SSG format one (19). There was a significant association between the SSG formats and the amount of turning conducted (χ2(3) = 12.58, *p* < 0.05). The players conducted a large amount of dribbles when participating in SSG format three (45); however, there were considerably fewer dribbles during SSG format one (20). There was a significant association between the SSG formats and the amount of dribbling performed (χ2(3) = 9.82, *p* < 0.05). The players conducted a large amount of shots when participating in SSG format four (45), whereas, when participating in SSG format one the players took much fewer shots (18). There was a significant association between the SSG formats and the amount of shots taken (χ2(3) = 14.27, *p* < 0.05). SSG format four provided the most goals (40), whilst SSG format one provided the fewest goals (15). There was a significant association between the SSG formats and the amount of goals scored (χ2(3) = 17.30, *p* < 0.05) (Table 3).

The players performed a large amount of overlaps when participating in SSG format three (39), whereas only 12 overlaps were conducted when playing SSG format one. There was a significant association between the SSG formats and the amount of overlaps performed (χ2(3) = 20.51, *p* < 0.05). There were 10 one-two situations observed when the players participated in SSG format four, whilst there were only 4 one-two situations when the players participated in SSG format three. However, there was no significant association between the SSG formats and the amount of one-twos conducted (χ2(3) = 3.23, *p* > 0.05). 1v1 situations were most commonly observed when the players participated in SSG format one (17), with the fewest 1v1 situations being observed in SSG formats three and four (both 9). There was no significant association between the SSG formats and the amount of 1v1 situations (χ2(3) = 3.98, *p* > 0.05) (Table 4).

## 4. Discussion

The purpose of this study was to investigate the influence of the number of goal-posts and the positioning of goal-posts used within SSG on the frequency of technical actions and offensive scenarios performed by players within soccer. The findings suggest that the use of different formats of SSG can influence the frequency of turns, dribbles, shots, goals and overlaps performed by prepubescent soccer players. It was hypothesized that different formats of SSG will lead to different frequencies of technical actions and offensive scenarios being performed by prepubescent soccer players and this can be supported for some of the technical actions and offensive scenarios. Due to the significant associations, it could be suggested that SSG formats explored within this investigation can encourage the use of particular behaviours during prepubescent soccer. The findings support previous research that different SSG conditions elicit different technical responses [9,34]. The findings of the current study support the work of Aguiar et al. [30] who concluded that SSG can be manipulated by the coach in order to produce different technical demands.

The technical action the players performed most within the SSG was passing. This is in support of Taylor et al. [35] who found each player (regardless of position) performed more passing than any other technical skill. There were 803 passes attempted across the four SSG formats, with format one having the most passes attempted (232 passes) and format four having the least passes performed (168 passes). SSG format one was a standard game with the goal-posts positioned centrally. The attacking team in this format would regularly pass the ball to team-members in wide areas or those who were supporting in a deeper position. This enabled players on the attacking team to receive the ball under less defensive pressure and to keep possession of the ball for the attacking team. The least amount of passes were performed in format four and this may be attributed to having two goals to attack that could be scored through the front and back of the goal. This enabled greater opportunities for the players to shoot and therefore the participants may have chosen this technical action rather than passing to a team-member. Almeida et al. [34] concluded that the amount of time spent on deliberate practice could be an important component of soccer development programmes. It could be suggested the use of format one produces the greatest amount of passing practice in a game-like environment, and would therefore be most beneficial to a coach who is aiming to encourage a high amount of passing within a SSG.

One interesting finding that emerged from the data was that the highest percentage of unsuccessful passes occurred in SSG format four (25.0%). This was in comparison to the lowest percentage of unsuccessful passes that occurred in SSG format three (14.8%). Format four had two goals positioned in wider areas compared to format three which had one goal positioned centrally. This finding is different to that reported by Almeida et al. [7] who found that when players participated in SSG with two goals they experienced a lower risk of losing possession through opponents’ interceptions or turnovers. Accounting for this difference may be attributable to a number of factors: (1) variability within the passing ability of the participants; (2) the defensive organization, co-operation and tactical decision-making of the team not in possession; and (3) the attacking intent and decision-making of the team in possession. Nevertheless, the difference in the findings suggest further research is required to examine in more detail the extent to which manipulating the number and positioning of the goal-posts simultaneously influences the inter-relationship between the defensive and attacking tactical decision-making of both teams.

The prepubescent players performed the largest amount of turns when playing SSG format two (45), whilst turning was not frequently performed during SSG format one (19). There was a significant association between the SSG formats and the amount of turning conducted. SSG format two has the goal-posts positioned in each corner of the pitch, meaning that the attacking team has two potential areas to attack. As the team aim to attack one of the goals, the defensive team will move towards this goal in an attempt to prevent the attacking team from scoring. As this happens, the attacking player in possession of the ball may turn and move towards the other goal, where there will be less defensive pressure. This game situation will occur frequently in SSG format two therefore this format may be a valuable game for developing the skill of turning within prepubescent soccer players. Clemente et al. [27] stated that using an end line as a scoring method may promote the exploitation of attacking players in different zones. Although the current study did not use an end line as a scoring method, it appears that the players during SSG format two (two goals to target) were using the turn to utilize supporting players in other areas of the pitch. This game may be important for coaches to implement as turning has previously been identified as a key technical action within soccer [36].

The prepubescent players performed the greatest amount of dribbles when participating in SSG format three (45); however, there were considerably fewer dribbles during SSG format one (20). There was a significant association between the SSG formats and the amount of dribbling performed. In SSG format three the goal-posts were positioned infield by 9.14 m/10 yd and the attacking team could only score by passing/shooting the ball through the back of the goal. During this SSG format the researchers regularly observed that when the attacking team moved the ball into the wide areas of the pitch, players would regularly dribble the ball towards the end line of the pitch and then attempt a pass/cross to a team-member who would attempt to score. The central area near the goal would usually have a cluster of defensive players, so the wide areas provided a space for attacking players to dribble into. Clemente et al. [27] found clusters of defensive players when teams had two goals to defend in SSG. It appears that players within the current study adopted a similar tactic when defending a single goal that was positioned in-field. There was a similar trend for dribbling observed between the present study and the Manchester United 4v4 pilot scheme conducted by Fenoglio [19]. Within the current study and the Manchester United 4v4 pilot scheme study, the 4-goal game (SSG format two) produced more dribbling actions (37 and 45, respectively) than the 2-goal game (SSG format one) (20 and 39, respectively). However, unlike the present study that used the same eight participants for all data collection, data collection from the Manchester United 4v4 pilot scheme involved players from numerous academies [19]. Caution should therefore be taken when comparing the results from these two studies.

The prepubescent players conducted the highest amount of shots when participating in SSG format four (45). When participating in SSG format one the players only took 18 shots. There was a significant association between the SSG formats and the amount of shots taken. It appears that coaches could apply SSG format four to training sessions to encourage shooting within prepubescent soccer players. However, due to the absence of goalkeepers in the current study, coaches should be aware that shooting within format four may not demand the same decision-making skills that are required in a real game situation [37]. Travassos et al. [28] concluded that further research was required within SSG to investigate the presence or absence of a goalkeeper. It would be suggested that various factors including technical actions and offensive scenarios could be altered with the presence of a goalkeeper, so this is a valuable area to investigate.

The players performed a large amount of overlaps when participating in SSG format three (39), whereas only 12 overlaps were conducted when playing SSG format one. There was a significant association between the SSG formats and the amount of overlaps performed. It appears that format three created higher numbers of defensive uncertainty and gained more advantages for the attacking team [14]. Format four produced the second highest number of overlapping runs (20). For SSG formats three and four the goal-posts were positioned 9.14 m/10 yd in-field. The positioning of the goal-posts in this way may have encouraged the players to progress into advanced positions on the pitch when not in possession of the ball, in order to potentially receive the ball in a more advanced position. Coaches should consider using SSG format three when aiming to encourage prepubescent players to develop overlapping runs as a tactical strategy. However, the current study recorded all overlapping runs, without reference to whether the player making the overlapping run received the ball, or if the team gained an advantage from the overlap such as an attempt at goal, or completion of a penetrating pass. To enable a more detailed insight into the effect of overlapping runs using format three, future research should also record subsequent team actions following an overlapping run.

The main limitation of the study is related to the possible impact of fatigue on soccer performance during the different SSG formats [21]. The participants were aware that they would play 2 × 10 min games of a certain SSG format during one session. This could have led to participants regulating effort in the first 10 min game, so that they would not be fatigued for the second 10 min game [38]. Conversely, the participants may have expended a large amount of energy during the first 10 min game and therefore experienced the impact of fatigue during the second 10 min game. Future studies should consider the design of the session to limit the potential impact of fatigue on practical performance.

## 5. Conclusions

The aim of this study was to investigate the influence of the number of goal-posts and the positioning of goal-posts used within SSG on the frequency of technical actions and offensive scenarios performed by prepubescent players within soccer. Due to the significant associations observed, it could be suggested that SSG formats explored within this investigation can encourage the use of particular behaviours during prepubescent soccer. It should be noted that a limitation of the one sample chi-squared test is that it is not able to indicate the size of the statistical difference between the different SSG formats. It is suggested coaches should consider using a variation of the number and positioning of goal-posts in SSG as an effective training tool in the development of prepubescent soccer players. This will enable coaches to vary the focus of sessions, and develop specific technical and tactical actions within a situation similar to that of real match-play. One of the main limitations of the current study is the duration of each SSG (20 min for each format). Analysis of a greater amount of time for each SSG format (e.g., 6 × 10 min) may provide a more representative profile of the performance for each format.

## Figures and Tables

**Table 1:** Action variables and operational definitions.

Category	Action Variable	Operational Definition
Technical Skills	Pass forwards	A pass when the ball is played towards the opponent’s goal
	Pass sideways	A pass when the ball was neither played towards the goal that the player is defending or towards the opponent’s goal
	Pass backwards	A pass when the ball is played towards the goal that the player is defending
	Successful pass	A pass that is received by a team-member who then has controlled possession of the ball
	Unsuccessful pass	A pass is attempted but a team-member fails to receive the pass or have controlled possession of the ball following the pass
	Penetrating pass	A pass that breaks a line of defence
	Turning	Using the foot to change direction of the ball in an attempt to exploit an opportunity in another area of the pitch
	Dribbling	Manoeuvering the ball using three or more touches through the use of technical actions whilst travelling with the ball
	Shot	When an attacker kicks or heads the ball in a deliberate attempt to score a goal
Other	Goal	When the entire ball crosses the whole line between the goalposts
Offensive scenarios	Overlap	In an attempt to receive the ball, an attacking player makes a run forward on the outside of the player in possession of the ball
	One-two	A player passes to a team-member and receives the ball back after making a run to the opposite side of the defender
	1v1	An isolated situation between one attacker and one defender with a deliberate attempt from the attacker to run or dribble with the ball past the defender

**Table 2:** Passing actions for each SSG format.

Passing Action	Format One	Format Two	Format Three	Format Four
Successful pass forwards	78 (33.6)	45 (21.0)	60 (31.7)	52 (31.0)
Successful pass sideways	27 (11.6)	55 (25.7)	42 (22.2)	23 (13.7)
Successful pass backwards	73 (31.5)	60 (28.0)	52 (27.5)	42 (25.0)
Penetrating pass	10 (4.3)	6 (2.8)	7 (3.7)	9 (5.4)
Unsuccessful pass forwards	32 (13.8)	42 (19.6)	17 (9.0)	32 (19.0)
Unsuccessful pass sideways	10 (4.3)	3 (1.4)	6 (3.2)	5 (3.0)
Unsuccessful pass backwards	2 (0.9)	3 (1.4)	5 (2.6)	5 (3.0)

**Table 3:** Technical actions for each SSG format.

Technical Action	Format One	Format Two	Format Three	Format Four
Turning	19	45	25	29
Dribbling	20	37	45	32
Shot	18	23	32	45
Goals	15	17	20	40

**Table 4:** Offensive and defensive scenarios for each SSG format.

Offensive Scenario	Format One	Format Two	Format Three	Format Four
Overlap	12	15	39	20
One-two	5	7	4	10
1v1	17	10	9	9
